# Impact of an antimicrobial stewardship program in the antimicrobial-resistant and prevalence of *clostridioides difficile* infection and amount of antimicrobial consumed in cancer patients

**DOI:** 10.1186/s13104-020-05085-3

**Published:** 2020-05-19

**Authors:** Masoud Mardani, Sara Abolghasemi, Shiva Shabani

**Affiliations:** Infectious Diseases and Tropical Medicine Research Center, Shahid Beheshti University of Medical Sciences, Tehran, Iran

**Keywords:** Antimicrobial stewardship (ASP) intervention, *Clostridium difficile* infection (CDI), Antimicrobial resistance, Meropenem, Vancomycin

## Abstract

**Objective:**

The The impact of a hospital antimicrobial stewardship was determined on antimicrobial-resistant, *Clostridioides difficile* rates and the amount of antimicrobial consumed in cancer patients.The intervention effects of antimicrobial stewardship (ASP) plans in 2017–2018 and 2018–2019 were respectively evaluated among hematology/oncology and bone marrow transplant patients in Ayatollah Taleghani University Hospital, Tehran, Iran. In this interventional quasi-experimental study, the ASP repository was utilized to capture four survey questions encompassed in these immunocompromised patients: amount of antibiotics (meropenem and vancomycin) consumption gr-year, the number of positive *Clostridioides difficile* infection and multidrug-resistant positive cases in blood cultures.

**Results:**

The number of MDR cases in the periods of 2017–2018 and 2018–2019 were 145 and 75, respectively (p = 0.011). A significant reduction in all positive blood cultures from 2017–2018 to 2018–2019 was found (p = 0.001). 574 patients admitted to our hospital in these periods of 2017- 2018 and 2018- 2019were assessed for MPM and VMN use. The amounts of MPM prescriptions in 2018–2019 was significantly decreased from 22464 to 17262 g (p = 0.043). The significant reduction in antibiotic consumption, MDR organisms, and CDI can highly promote patients’ health and decreasing medical costs and long-term defects for patients.

## Introduction

The inappropriate or overuse antibiotic usage, especially broad-spectrum agents results in the emergence and spread of antibiotic-resistant bacteria, which lead to increased morbidity, inpatient stays, and higher mortality [1–3].

Increased multidrug resistance (MDR) organisms and *Clostridium difficile* infections have been involved in solid-organ transplant (SOT) recipients and have been associated with significant graft loss and mortality [4]. Rates of bacterial infections in SOT recipients range from 21% to 68%, depending on transplant type and immunotherapy [5, 6]. Similarly, the incidence of *Clostridioides difficile* infection (CDI) in this population is increasing and characterized by more severe disease, frequent recurrences, and graft loss [4]. There are limited treatment options for MDR organisms, and antibiotic exposure remains the principal risk factor for CDI. Therefore, transplant providers, patient safety plan and programmatic interventions focusing on prevention are an emerging problem [4, 7].

Over the past few decades, published literature related to the safety and efficacy of antibiotic stewardship strategies in high-risk immunocompromised hosts such as SOT recipients and those with hematologic malignancies exposed to cytotoxic chemotherapy and prolonged neutropenia is limited [8]. Malignancies need efficient antibiotics to treat infectious complicated states in various degrees of immunosuppression and chemotherapy. In leukemia and malignant hematology and stem cell transplant (HSCT) patients vancomycin-resistant enterococcus (VRE) bloodstream infections were associated with a 2.9-fold increase in mortality and morbidity compared in patients without VRE infections. Established ASP in the HSCT population such as prior approval and post prescription review is feasible, effective, and safe in this patient population [9].

These resistant microorganisms could rise in immunocompromised conditions, like individuals who have undergone organ transplantation leads to further spread of MDR [10].Among adult hospital ASPs, studies have indicated a reduction in the hospital length of stay in ICU, a decrease in *C. difficile* rates, and a development in antibiotic susceptibilities of common pathogens [11, 12].

The objective of this study was to determine the impact of a hospital ASP on antimicrobial-resistant, *Clostridium difficile* rates and the amount of antimicrobial consumed meropenem (MPM) and vancomycin (VMN) in cancer patients.

## Main text

### Materials and methods

#### Antimicrobial stewardship program (ASP) setting and population

In this interventional quasi-experimental study we evaluated the effects of ASP plans among hematology/oncology and bone marrow transplant patients in Ayatollah Taleghani University Hospital, in Tehran, Iran. All patients aged > 18 years admitted through the medical wards.

Based on the approved guidelines of health and medical education in Iran in May 2018 rational usage of antibiotics in hospitalized patients should be prescribed according to the instruction of the infectious diseases specialists. Therefore the ASP repository was utilized to capture four survey questions encompassed in these immunocompromised patients: measuring the amount of meropenem and vancomycin (gr-year) usage, amount of antibiotics (MPM and VMN) consumption (gr-year), number of positive *Clostridioides difficile* infection and MDR positive cases in all blood cultures were compared during two time periods, the first period was considered as the 12 months before the ASP implementation, while the second period was the first 12 months that the ASP was in place. Also, continuous educational programs were provided for all nurses and physicians employed in different wards of the hospital. Besides, an interdisciplinary ASP team with an infectious disease specialist and a pharmacist performed weekly and evaluated all the data collection and the patient’s electronic medical record, afterward distributed some of main determinations/counseling with specialists to use of a sole antibiotic with a less or more dose, and sent feedback to providers. The study performed in periods of 2017–2018 and 2018–2019 and was not limited to a specific season to cause any disruption. Table 1 summarizes all detailed data in this study.

**Table 1:** A summary of antibiotic prescriptions, MDR and CDI positive cases issued for the studied patients in periods of 2017–2018 and 2018–2019

Characteristics	Period 2017–2018 2018–2019 (without ASP) (with ASP)		*P* value
Antibiotics (g)			
MPM^a^	22464	17262	0.043
VMN	15180	14400	0.07
MDR^a^ (in blood cultures)	145	75	0.011
CDI positive cases inwards (n %)			
Bone marrow transplant^a,b^	5/43 (11.6)	1/37 (2.7)	0.004
Oncology	4/47 (8.5)	4/51 (7.8)	0.09

^a^Values in the same row followed by different letters (a, b) are significantly

^b^Bone marrow transplantation

#### CDI case definition

A patient was diagnosed with CDI when he or she had diarrhea (three or more unformed stool movements within 24 h) and tested positive in an approach included an ELISA A + B kits (Abnova, Catalog Number KA3202) [13].

#### Data collection

The ASP team inspected the primary instructions and applied usage arrangements of MPM and VMN according to the antimicrobial susceptibility patterns of clinical isolates. Our ASP team was including a specialist in infectious diseases, cancer, and clinical microbiology. The daily and weekly usage arrangements of MPM and VMN was based on the ASP protocol and determined according to the antimicrobial susceptibility patterns of clinical isolates or type of antibiotic-resistant infections and misusing risk.

Antimicrobial stewardship best practices guiding principles include the recommendations on multidrug resistance bacteria in bone marrow transplant recipients and the Urinary Tract Infection (UTI) Guideline from the American Society were performed where applicable. As a brief, the UTI was recognized from the clinical charts of all patients with substantial quantities of pathogens isolated in blood cultures and was confirmed by testing their records to find the same pathogen in the urine and blood cultures. The diagnosis of respiratory tract infection was performed by fever (T > 38.0 °C) or hypothermia (T < 35.5 °C), leukocytosis or leukopenia, and positive tracheal culture. [14].

**Fig. 1:**
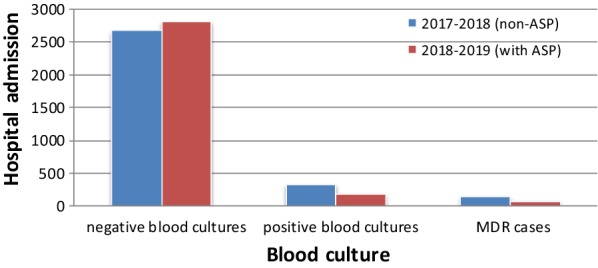
The admission number of positive and negative blood cultures in different wards of Ayatollah Taleghani Hospital during periods of 2017–2018 and 2018–2019

The ASP repository was utilized to capture the data collected on each patient review including antibiotic(s) prescribed, the dose of antibiotic(s) and clinical data. Bacterial blood infection was assessed by checking their records to find the positive Gram staining results and detection of MDR bacteria (Fig. 1). Consumption of VMN and MPM was expressed as the defined gram per year for all 574 patients before and after the ASP. The costs of antimicrobial agents were not standardized, because they changed significantly during the study period. Our finding that enhanced antibiotic stewardship, including the restriction in the use of high-risk broad-spectrum antibiotics, including MPM and VMN with prescribing Tazosin, aminoglycosides or fluoroquinolones were prescribed in some patients, which was associated with a reduction in the number of CDI and MDR cases.

The intervention was composed of demanding confirmed prescription that diarrhea has encountered CDI clinical criteria, ASP pre authority, and oral practitioner feedback. *C. difficile* specimens were evaluated using ELISA assay kit by the clinical microbiology laboratory.

### Statistical analysis

The data were statistically assessed using SPSS software package. The independent t-student test and Chi square (χ2) or Fisher’s exact tests were performed to assess between-group differences. The significant level was set at p < 0.05.

### Results

We collected the number and percent of fecal specimens in the various hospital wards of oncology and transplant populations. The absolute number of CDI was reduced from 11.2% patients per year in bone marrow transplantation wards in May 2017–May 2018 (period 2) to 2.7% per year in May 2018–May 2019 (period 1) (P = 0.004) (Table 1). The number of MDR cases in the periods of 2017–2018 and 2018–2019 were 145 and 75, respectively (p = 0.011). A significant reduction in positive blood cultures from 2017–2018 to 2018–2019 was found (p = 0.001).Also, the amount of MPM prescriptions in 2018–2019 was significantly decreased from 22464 to 17262 g (p = 0.043).

### Discussion

It is worth recalling that the implementation of ASP guidelines could promote the management of patients in different wards of hospitals [15, 16].

The extensive application of ASPs in oncology and/or transplant patients has been associated with decreasing the inappropriate use of antimicrobial and cost reduction without resulting in harm in many countries [7, 17–19].

In a survey estimating ASPs at United States transplant centers, guideline development was a highly utilized antimicrobial stewardship intervention, occurring in 76 and 71% of hematopoietic stem cell transplant and solid organ transplant centers, respectively [7, 8]. We couldn’t assess the economic benefit of this culture-guided ASP, because the total cost of drugs was improved from 2017 to 2019. The results indicated that reduced the treatment amount of MPM successfully (P = 0.043). Transplant patients use the wide spectrum antibiotics and recent transplant cohort studies revealed significantly lower susceptibilities of common Gram-negative organisms isolated from their urine and blood [20]. In this study, the outcomes of the ASP method to control the antibiotic use VMN in healthcare settings were not improved (P = 0.07). This stewardship-discordant has been reported in another similar study that more than 40% of SOT patients were categorized as being discordant with stewardship principles [5, 21]. Results of other extensive plans showed that the ASP utilization can significantly reduce the true antimicrobial drug with an appropriate antibiotic dose, and duration of MPM, VMN and diminish the number of positive cultures [22–24].

Other similar studies in Singapore, Iran, and the US in hospitalized children and women showed that the proper prescription of carbapenems using the ASP was increased significantly, also proved that the ASP protocol can be successfully applied to promote quality of care of hospitalized patients [22, 25, 26].

Also, a relatively high infection rate of *Clostridioides difficile* was observed in our patients and found to exist in their environment. The incidence of CDI among Iranian cancer patients in large teaching hospitals was estimated to be 10.4%, which was lower than what was reported in the cancer hospitals in Spain, Brazil and China with findings of 17.3%, 48.3%, 15%, respectively [27]. Similar to the results of other studies in Canada, the US, and Iran the rates of CDI and MDR positive cases are decreased significantly in our patients (P = 0.004 and 0.011) respectively. [5, 23, 28].

Hematological malignancies and HSCT double CDI risks due to exposure to healthcare facilities, disruption of the microbiome from broad-spectrum antimicrobials and chemotherapy, and are associated with higher mortality [9].

The strategy of audit and feedback or post-prescription review is effective in reducing antimicrobial consumption in patients with hematological malignancies [9].

Our finding that enhanced antibiotic stewardship, including the restriction in the use of high-risk broad-spectrum antibiotics, including fluoroquinolones, clindamycin, and cephalosporins was associated with a reduction in the number of CDI and MDR cases.

Reduced antibiotic prescription of MPM and VMN and prescription of other antibiotics including aztreonam, daptomycin, ertapenem after the utilization of the ASP method was assessed by Malani et al. [29].

However, the pattern and route of antibiotic therapy were not considered in our study which may be a practical issue in further studies, especially in the immunocompromised population. The aim of future ASPs should include the avoidance of high-risk antibiotics which is coupled with infection control and lower rates of resistance.

### Conclusions

The presence of a strict ASP in a hospital is needed to guarantee optimal care for patients. This study compares the clinical impacts of the ASP with the conventional method and reports an antimicrobial restriction policy for MPM and VMN antibiotics. The rates of CDI and MDR positive cases are decreased significantly and a significant reduction was found in positive blood cultures from 2017–2018 to 2018–2019. ASP can reduce an appropriate antibiotic doses of MPM and VMN and antimicrobial restriction policy reduced the treatment amount of MPM successfully.

## Limitations

The pattern and route of antibiotic therapy were not considered in our study which may be a practical issue in further studies, especially in the immunocompromised population. Further expansion of limitations were limitations of retrospective studies, confounding over time and lack of measurement of other antibiotic agents. Also, the economic and long-term effects of this stewardship were not evaluated in this study. This study was done at one institution with an ASP that focuses on cancer patients and our finding didn’t show the economic benefits of prior antimicrobial approval by a dedicated ASP team among immunocompromised patients.

## Data Availability

All data generated or analyzed during this study are included in this published.

## Abbreviations

ASP: Antimicrobial stewardship program
MPM: Meropenem
VMN: Vancomycin
MDR: Multidrug-resistant
ICU: Intensive care unit
SOT: Solid-organ transplant
CDI: *Clostridium difficile* infection
